# “A herder’s duty is to think”: landscape partitioning and folk habitats of Mongolian herders in a mountain forest steppe (Khuvsugul-Murun region)

**DOI:** 10.1186/s13002-019-0328-x

**Published:** 2019-11-20

**Authors:** B. Gantuya, Á. Avar, D. Babai, Á. Molnár, Zs Molnár

**Affiliations:** 1 Botanic Garden and Research Institute, Mongolian Academy of Sciences, Ulaanbaatar, 13330 Mongolia; 2 Department of Mongolian and Inner Asian Studies, Eötvös Loránd University, Budapest, H-1088 Hungary; 3 Institute of Ethnology, Research Centre for the Humanities, Hungarian Academy of Sciences, Budapest, H-1097 Hungary; 4 Szeged, Hungary; 5 Institute of Ecology and Botany, MTA Centre for Ecological Research, Vácrátót, H-2163 Hungary

**Keywords:** Landscape partitioning, Mongolian herders, Continental forest steppe, Traditional ecological knowledge, Folk habitat, Landscape ethnoecology, Rangeland, Pasture, Herders’ knowledge

## Abstract

**Background:**

Traditional habitat knowledge, like the classification of folk habitats and how people partition their landscape into habitats, is an emerging but still understudied part of traditional ecological knowledge. Our objectives were to reconstruct the folk habitats and the partitioning of the landscape into these folk habitats by Mongolian herders in Northern Mongolia and to compare it with other Northern Hemisphere boreal-temperate classifications.

**Methods:**

The study area is located in Seruun Gilad (Khuvsugul province) and belongs to the mountain forest steppe of the Khangai region (dominated by meadow steppes and larch forests). Most herder families use the area for summer pasturing. Data collection was based on indoor and outdoor, structured and semi-structured interviews and interviews during landscape walks and participatory fieldwork. We interviewed 20 people using 76+ photos of plant species and 25+ photos of habitats and asked them to name and describe the habitats and describe the habitat preferences of the species.

**Results:**

Mongolian herders distinguished at least 88 folk habitat categories and knew well the habitat preferences of the 76 plant species. They argued that a herder has to be observant of nature. The habitat classification was moderately lexicalized, with many descriptive expressions. Most habitats (77%) belonged to the meso-scale, while macro-scale habitats (like *taiga*, *Gobi*) and micro-scale habitats (like *marmot burrow*, *top of the tussock*) were few. Habitat names did not reflect directly the usefulness of the habitat. Classification was multidimensional; key dimensions were geomorphological and edaphic. There were some species (e.g., *botyuul*, *hyag*, *shireg*) and species groups (*hot plants*, *leafy plants*) that were often used to describe habitat types.

**Conclusions:**

Landscape partitionings in the Northern Hemisphere differed considerably in the importance of various dimensions used, with edaphic, geomorphological, hydrological, and dominant species-based dimensions having higher importance, while land use, successional, and zoological dimensions having lower importance. We argue that conducting research on folk habitats will contribute to a deeper understanding of how nature is perceived by locals and to a more efficient management of the Mongolian pastures.

## Background

Sustainable use of natural resources demands a deep understanding of the managed ecosystems [1]. For example, rangelands utilized by nomadic, transhumant, or sedentary herders may degrade if governance and management are not adapted to the carrying capacity of the forage resources [2–5]. Traditional pastoral communities all over the world have a long-term, place-based understanding of the spatio-temporal patterns of their pasture resources (e.g., [6–9]). This knowledge is dynamic and has been transmitted through generations and adapted to changing socio-ecological conditions [10]. A significant part of this knowledge refers to the living components of the local environment pastoralists live in.

Ethnobiologists seek to understand how different human communities perceive, classify, and mentally process the living world and how people then apply that knowledge [11]. This knowledge is often called traditional ecological knowledge [1]. Ethnobiologists have traditionally focused on the folk classification and traditional use of plants and animals [12]. In the last decades, more and more studies are available on the habitat and vegetation-related traditional knowledge of local traditional communities, including herders, farmers, fishers, and hunters (e.g., [13–16]). Johnson and Hunn [15] introduced the term landscape ethnoecology which focuses on the ecological features of a landscape (e.g., ecotopes, habitats, vegetation types, and other landscape elements) and aims to understand how the living landscape is perceived, named, imagined, classified, and managed by people who live in it. Landscape elements, including habitat patches, are generally less discrete elements of the environment than plant and animal individuals. However, locals have a deep knowledge of these landscape elements including their characteristic species, structure, and function. In case of habitats, only variants exist in nature, which can be arranged along continua and around prototypes in classifications ([17], sensu [18]). Landscape elements have a diverse terminology in ethnobiology: ecotope, habitat, kind of place, and biotope [19]. We follow Molnár [20] and use the term habitat that includes all living creatures on a piece of land with its soil, bedrock, and hydrology. A habitat is mostly defined by its vegetation and soil and is more or less a synonym of ecotope.

Evidence shows that people in traditional communities tend to distinguish many habitats in a landscape; the average number of basic-level categories seems to be around 25 [21], whereas synonymous names are not rare. Locals distinguish habitats at different spatial scales. While folk plant classifications are most often strongly hierarchical [12], folk habitat types are usually ordered into less hierarchical partitionings and are often multidimensional [21]. A landscape partitioning is multidimensional if the partitioning uses several distinct sets of salient environmental features to define habitats, and habitats cannot be arranged in a unidimensional system (cf. [22]).

Whereas traditional ecological knowledge of African pastoralist communities is well-documented (e.g., [6, 8, 23–26]), herder communities in northern regions are less often studied. One of the most famous groups of pastoralists is the nomadic and semi-nomadic Mongolian pastoralists. The herding tradition of Inner-Asian nomads dates back at least 3000 years [27]. Nomadic knowledge of nature is passed on from generation to generation but adapted to the changing conditions [3, 28]. Herder families still practice many old traditions, including using yurts (*ger* in Mongolian) and utilize pastures in a seasonal rotation to avoid overgrazing [29]. Roughly 30% of Mongolians are pastoralists, and at least half of the Mongolians still depend on the pastoral economy for their livelihood [3]. Mongolian herders use their traditional ecological knowledge of seasonal changes, dynamics of pastures, etc. to determine the grazing areas they will move to. This understanding of nature is especially important during the extreme winter conditions (*dzud*), which can kill many of their animals [30, 31]. Mongolians possess a detailed knowledge of pasture forage species [32, 33] and pasture degradation [34, 35] but also have a rich knowledge of traditional herbal plants and medicinal animals [28, 36, 37]. Ecological knowledge of herders seems to be fairly independent of the scientific botanical or ecological knowledge though knowledge has been significantly affected by schooling and recently by the Internet and social media [38]. For the moment, we are not aware of any detailed documentation of the folk habitats Mongolians recognize, name, and use in their everyday activities or any traditional landscape partitioning of a concrete Mongolian landscape.

Traditional habitat knowledge has been documented in an increasing number of cases in temperate and boreal regions of the Northern Hemisphere, for example, among the Gitskan and Kaska First Nations in Canada [39, 40], among the Alleutais in the French Alps [13, 41], among herders in the Hortobágy forest steppe in Hungary [16, 20], Sami herders in Scandinavia [7], and among the Csángós living in the Carpathian Mountains in Central Europe [22, 42]. Comparison of these classifications and partitionings may help us better understand how people perceive landscapes. However, comparing folk habitat classifications seems to be more difficult than comparing plant folk classifications, as habitat knowledge is more implicit and more variable, and often more difficult to elicit [20, 43, 44], and we also lack a globally standardized habitat taxonomy.

In this paper, our objectives are to reconstruct the folk habitats and the partitioning of the landscape into these folk habitats by Mongolian herders in a forest steppe area in Northern Mongolia and to compare it with other Northern Hemisphere boreal-temperate (and some tropical) classifications.

## Materials and methods

### The landscape

**Fig. 1:**
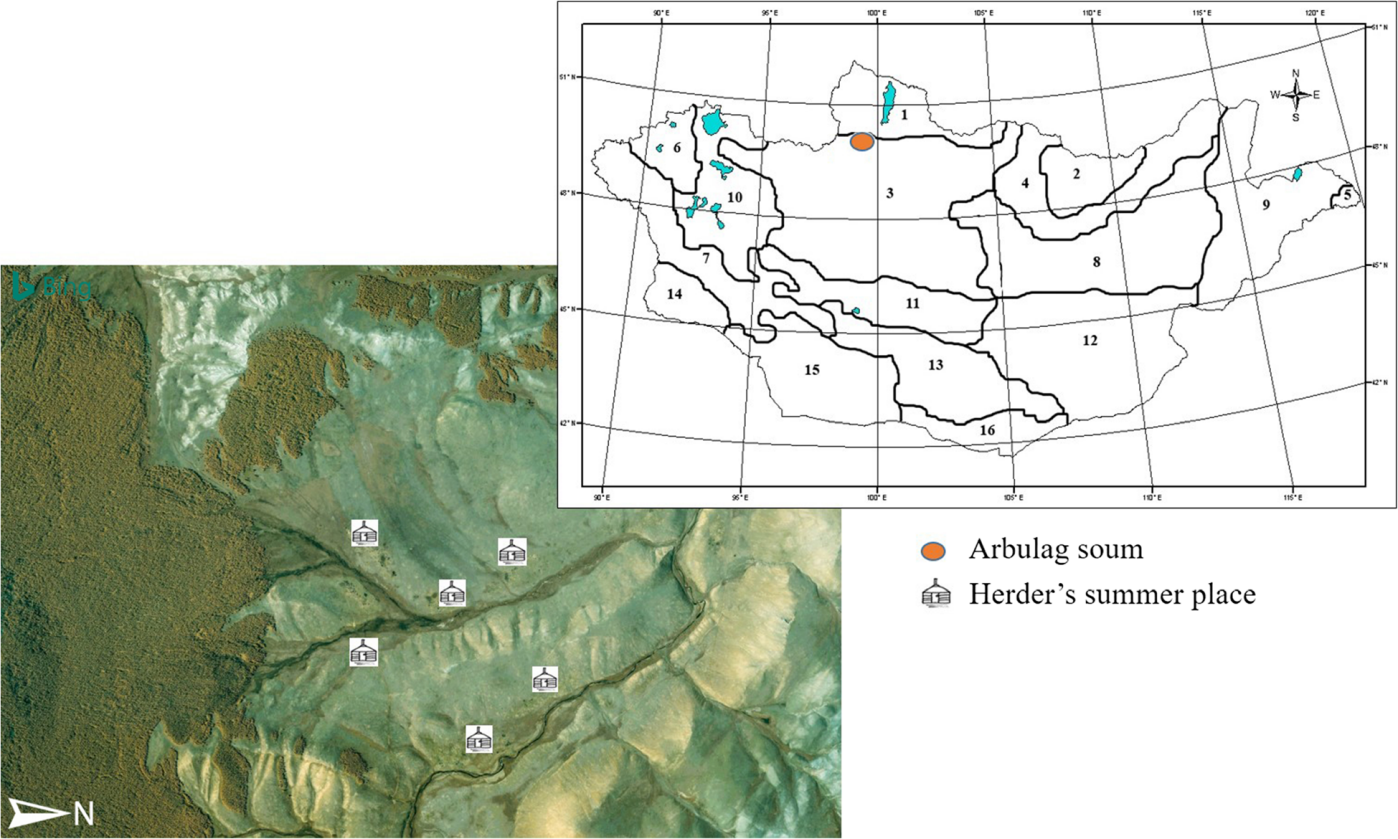
The study area is located in the mountain forest steppe of Khangai region (no. 3), in Arbulag soum, Khubsugul province, Northern Mongolia (source: V.I. Grubov, 1982)

The study area is located in Seruun Gilad, in Arbulag soum (district) at the border to Bayanzurkh soum, in Khubsugul province, Northern Mongolia (coordinates N 50° 22′ 47.8″, E 99° 31′ 52.1″) (Fig. 1). The area lies 1300–1900 m above sea level and has a diverse geomorphology with steep slopes and gentle hills, rock outcrops, and river floodplains. The area belongs to the mountain forest steppe of the Khangai region [45, 46], with relatively natural vegetation utilized by relatively traditional pastoralist communities. According to local herders, the area is a cold upland pasture area with patches of forest. It lies in the permafrost region of Mongolia. The climate is characterized by 250–450 mm annual precipitation; the average monthly temperatures range from + 12 °C in July to − 21 °C in January. The mean yearly temperature is − 4.5 °C, the absolute maximum is + 35 °C, and the absolute minimum is − 49 °C [47].

*Land use* is based on semi-nomadic livestock keeping. Summer campsites are set up along streams or on lower hills close to the streams. Various types of livestock including sheep, goats, yaks, cattle, and horses are the main source of living. There were 347,159 heads of livestock (184,545 sheep, 130,000 goats, 13,984 horses, and in total 18,421 cattle and yaks) in Arbulag soum in 2017 (which has 320,550 ha of pasture, and its total area is 352,921 ha [48]. In recent years, there has been an increased impact of livestock grazing and climate change in this area, affecting rangeland conditions, e.g., species composition, vegetation structure, and productivity [49]. General degradation of rangelands is present in the study area as well. Overgrazing and its consequences are related to the free-moving right of Mongolian herders and to the overcrowded livestock [35, 50].

Most families use the study area for summer pasturing, except one family who also has the winter pasture in the northern part of the area.

*Ethnic groups* living in the study area (in Arbulag and Bayanzurkh soums) are mainly Khotgoid, Darkhad, and Khalkh communities. These communities are mainly pastoralists; the Darkhad community is well-known and studied because of Shamanism [51]. Locals still possess a relatively traditional culture and a characteristic semi-nomadic lifestyle and customs, with rich folklore and religious life.

**Fig. 2:**
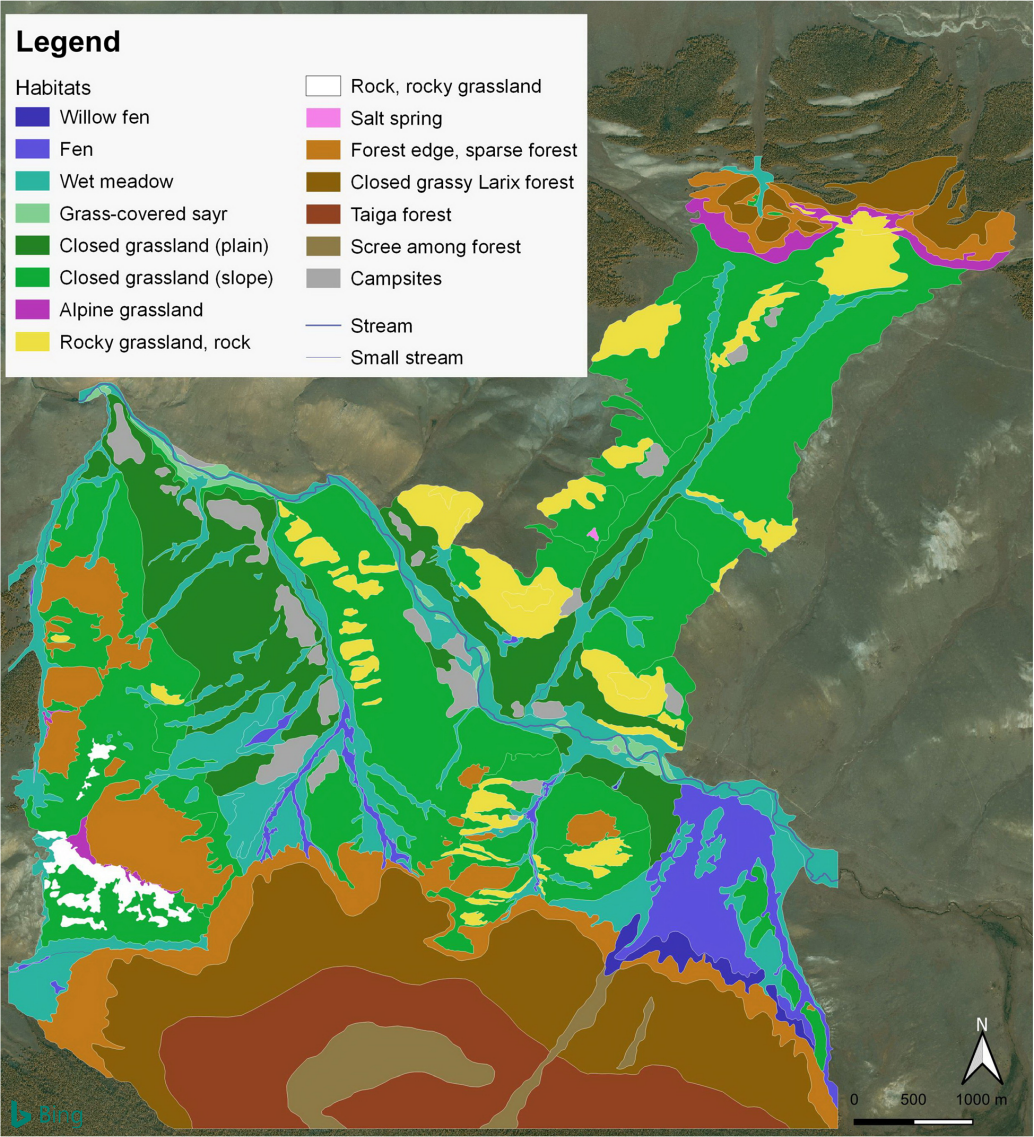
Vegetation (habitat) map of the study area (Seruun Gilad, Arbulag soum, Khuvsugul province, Northern Mongolia)

In 2017, we prepared a detailed vegetation (habitat) map of the study area based on the scientific (botanical) understanding of the vegetation but also taking herders’ perceptions into consideration when developing the habitat classification for the mapping (Fig. 2). A Bing map satellite image and QGIS 3.4.3. software were used. During mapping, all vascular plant species found were identified and listed; 179 species were collected and deposited in the herbarium of the Institute of General and Experimental Biology of the Mongolian Academy of Sciences, Ulaanbaatar.

The *vegetation* of the study area is mainly composed of mountain forest steppe, dominated by larch forests (dominant species: *Larix sibirica* Ledeb., *Vicia amoena* Fisch., *Trollius asiaticus* L., *Poa nemoralis* L., *Trisetum sibiricum* Rupr., *Polemonium chinense* (Brand) Brand, *Valeriana officinalis* L.), mountain meadow steppes (dominant species: *Festuca lenensis* Drob., *Echinops latifolius* Tausch, *Gentiana decumbens* L.fill., *Schizonepeta multifida* (L.) Briq.) and wet habitats (dominant species: *Carex duriuscula* C.A.Mey., *Primula nutans* Georgi, *Bistorta vivipara* (L.) S.F.Gray, *Parnassia palustris* L., *Juncus triglumis* L., *Gentiana pseudoaquatica* Kusn.) (Table 1).

**Table 1:** Main scientific habitat types of the study area and their dominant and characteristic plant species (ordered according to their dominance)

Scientific habitat name	Dominant and characteristic vascular plant species
Alpine zone (above the tree line, at the top of northern slopes)	*Dryas oxyodonta* Juz., *Poa altaica* Trin., *Kobresia sibirica* (Turcz. ex Ledeb) Boeck., *Gentiana algida* Pall., *Thalictrum alpinum* L., *Lagotis integrifolia* (Willd.) Schischk, *Poa sibirica* Roshev., *Hierochloa alpina* (Sw.) Roem. et Schult, *Saussurea schanginiana* (Wydl.) Fisch. ex Herd., *Erigeron flaccidus* (Bunge) Botsch., *Caragana jubata* (Pall.) Poir., *Potentilla fruticosa* L., *Ribes altissimum* Turcz. ex Pojark., *Rosa acicularis* Lindl., *Berberis sibirica* Pall., *Grossularia acicularis* (Smith) Spach, *Lonicera altaica* Pall. ex DC., *Pedicularis resupinata* L., *Pulsatilla flavescens* (Zucc.) Juz., *Saxifraga cernua* L., *Parnassia laxmannii* Pall. ex Schult., *Potentilla nivea* L., *Calamagrostis purpurea* (Trin.) Trin.
Taiga forests (closed, mossy)	*Vaccinium vitis-idaea* L., *Ledum palustre* L., *Rhododendron parvifolium* Adams, *Empetrum sibiricum* V.Vassil., *Thalictrum alpinum* L., *Pyrola rotundifolia* L., *Pyrola incarnata* (DC.) Freyn, *Linnaea borealis* L., *Juniperus sibirica* Burgsd., *Minuartia arctica* (Stev. ex Ser.) Aschers. et Graebn., *Arctous alpina* (L.) Niedz., *Campanula turczaninovii* Fed., *Equisetum palustre* L., *Lonicera altaica* Pall. ex DC., *Cacalia hastata* L., *Aconitum septentrionale* Koelle.
Light forests (grassy)	*Vicia amoena* Fisch., *Poa nemoralis* L., *Valeriana officinalis* L., *Androsace septentrionalis* L., *Aconitum barbatum* Pers., *Polemonium chinense* (Brand) Brand, *Bromopsis pumpelliana* (Scribn.) Holub., *Trisetum sibiricum* Rupr., *Pedicularis verticilliata* L., *Allium schoenoprasum* L., *Rosa acicularis* Lindl., *Sanguisorba officinalis* L., *Luzula rufescens* Fisch. ex E.Mey., *Hedysarum inundatum* Turcz., *Saxifraga sibirica* L., *Corallorhiza trifida* Chatel., *Actaea erythrocarpa* Fisch., *Deschampsia cespitosa* (L.) Beauv.
Forest fringes	*Pulsatilla flavescens* (Zucc.) Juz., *Pulsatilla turczaninovii* Kryl. et Serg., *Anemone crinita* Juz., *Trollius asiaticus* L., *Salix glauca* L., *Salix pseudopentandra* (B.Flod.) B.Flod., *Myosotis sylvatica* Ehrh. ex Hoffm., *Bromopsis inermis* (Leys.) Holub., *Poa nemoralis* L., *Dianthus superbus* L., *Potentilla fruticosa* L., *Senecio campester* (Retz.) DC., *Senecio integrifolius* (L.) Clairv., *Calamagrostis purpurea* (Trin.) Trin., *Trifolium lupinaster* L., *Aster alpinus* L., *Bistorta alopecuroides* (Turcz. ex Meissn.) Kom., *Festuca lenensis* Drob.
Mountain meadow steppes (including rocky grasslands)	*Patrinia sibirica* (L.) Juss., *Rhaponticum uniflorum* (L.) DC., *Echinops latifolius* Tausch, *Galium verum* L., *Gentiana decumbens* L.fil., *Koeleria cristata* (L.) Pers., *Veronica incana* L., *Festuca lenensis* Drob., *Agropyron cristatum* (L.) Beauv., *Rheum undulatum* L., *Erysimum flavum* (Georgi) Bobr., *Schizonepeta multifida* (L.) Briq., *Arctogeron gramineum* (L.) DC., *Artemisia dolosa* Krasch., *Artemisia frigida* Willd., *Dracocephalum foetidum* Bunge, *Thymus* sp., *Chamaerhodos altaica* (Laxm.) Bunge, *Chamaerhodos erecta* (L.) Bunge, *Goniolimon speciosum* (L.) Boiss., *Bupleurum bicaule* Helm
Meadows near streams (riparian zone)	*Bistorta vivipara* (L.) S.F.Gray, *Carex duriuscula* C.A.Mey., *Primula nutans* Georgi, *Primula farinosa* L., *Gentiana squarrosa* Ledeb., *Gentiana pseudoaquatica* Kusn., *Cirsium esculentum* (Siev.) C.A.Mey., *Pedicularis longiflora* J. Rudolph, *Lomatogonium carinthiacum* (Wulf.) Reichenb., *Parnassia palustris* L., *Carex pediformis* C.A.Mey., *Halenia corniculata* (L.) Cornaz, *Pedicularis flava* Pall., *Lloydia serotina* (L.) Reichenb., (*Gagea serotina* (L.) Ker Gawl.), *Ranunculus pseudohirculus* Schrenk, *Taraxacum* sp., *Juncus triglumis* L., *Triglochin palustre* L.
Fen meadows with or without willows	*Dracocephalum grandiflorum* L., *Caltha palustris* L., *Trollius asiaticus* L., *Carex ensifolia* (Turcz. ex Gorodk.) V.Krecz., *Carex microglochin* Wahlenb., *Luzula sibirica* (V.Krecz.) V.Krecz., *Kobresia sibirica* (Turcz. ex Ledeb.) Boeck., *Anemone crinita* Juz., *Eriophorum polystachyon* L., *Gentiana grandiflora* Laxm., *Potentilla fruticosa* L., *Phleum phleoides* (L.) Karst., *Poa pratensis* L., *Bistorta alopecuroides* (Turcz. ex Meissn.) Kom., *Viola uniflora* L., *Epilobium palustre* L., *Agrostis stolonifera* L., *Sanguisorba officinalis* L.
Temporarily flooded rocky vegetation in and near streams (*sayr*)	*Lagopsis marrubiastrum* (Steph.) Ik.-Gal., *Taraxacum* sp., *Hordeum brevisubulatum* (Trin.) Link, *Dracocephalum fragile* Turcz. ex Benth., *Papaver nudicaule* L., *Leymus paboanus* (Claus) Pilg., *Dracocephalum foetidum* Bunge, *Ranunculus natans* C.A.Mey., *Tragopogon trachycarpus* S.Nikit., *Panzeria lanata* (L.) Sojak, *Setaria viridis* (L.) Beauv., *Chenopodium album* L., *Amethystea coerulea* L., *Achnatherum splendens* (Trin.) Nevski, *Chamaerhodos erecta* (L.) Bunge, *Lappula intermedia* (Ledeb.) M.Pop., *Lophanthus chinensis* (Rafin.) Benth., *Leptopyrum fumarioides* (L.) Reichenb., *Poa subfastigiata* Trin., *Dontostemon integrifolius* (L.) C.A.Mey., *Orostachys spinosa* (L.) C.A.Mey., *Artemisia macrocephala* Jacq. ex Bess., *Artemisia mongolica* (Bess.) Fisch. ex Nakai, *Stipa glareosa* P.Smirn.
Screes among forest	*Caragana jubata* (Pall.) Poir., *Ribes altissimum* Turcz. ex Pojark., *Atragene sibirica* L., *Chamaenerion angustifolium* (L.) Scop., *Berberis sibirica* Pall., *Saussurea involucrata* (Kar. et Kir.) Sch.Bip., *Saussurea dorogotaiskii* Palib., *Elymus gmelinii* (Ledeb.) Tzvel., *Rhodiola quadrifida* (Pall.) Fisch. et Mey., *Rhodiola rosea* L., *Rosa acicularis* Lindl., *Actaea erythrocarpa* Fisch., *Cortusa altaica* Losinsk., *Woodsia ilvensis* (L.) R.Br., *Corydalis sibirica* (L.fil.) Pers., *Allium altaicum* Pall., *Potentilla fruticosa* L., *Orostachys fimbriata* (Turcz.) Berger, *Saxifraga spinulosa* Adams, *Spiraea flexuosa* Fisch. ex Cambess.
Meadow steppes (in valley bottoms)	*Smelowskia alba* (Pall.) Regel, *Potentilla conferta* Bunge, *Polygonum aviculare* L., *Astragalus galactites* Pall., *Artemisia dolosa* Krasch., *Artemisia borealis* Pall., *Oxytropis strobilacea* Bunge, *Hordeum brevisubulatum* (Trin.) Link, *Senecio integrifolius* (L.) Clairv., *Androsace lactiflora* Pall., *Carex duriuscula* C.A.Mey., *Bistorta vivipara* (L.) S.F.Gray, *Lioydia serotina* (L.) Reichenb., *Sanguisorba officinalis* L., *Stipa glareosa* P.Smirn., *Leymus paboanus* (Claus) Pilg., *Chenopodium* sp.
Disturbed and ruderal places	*Plantago major* L., *Chenopodium album* L., *Urtica angustifolia* Fisch. ex Hornem., *Lepidium ruderale* L., *Leptopyrum fumarioides* (L.) Reichenb., *Potentilla anserina* L., *Potentilla bifurca* L., *Artemisia glauca* Pall. ex Willd., *Artemisia macrocephala* Jacq. ex Bess., *Agropyron cristatum* (L.) Beauv., *Puccinellia macranthera* V.Krecz., *Taraxacum ceratophorum* (Ledeb.) DC., *Chenopodium prostratum* Bunge, *Lappula* sp., *Leonurus deminutus* V.Krecz., *Amaranthus retroflexus* L., *Rumex crispus* L.

*Larch forests* have a sparse shrub layer, even the non-grazed ones. In the inner—non-grazed—parts of the forests, forest-interior species dominate, typical species of pastures are scarce, the moss layer is deep and dense, and there are a large number of dead trees lying on the ground (standing dead trees are unexpectedly rare). Stumps (indicating wood harvesting) and livestock dung (indicating grazing) are rare (or there is none) in these deeper forest areas. Small forest patches have almost no forest-interior flora, and the ground is dominated by shade-tolerant grassland species or is bare (with larch leaf litter, the whole patch can be regarded as a forest fringe). *Forest fringes* are usually not sharp, but 20–100 m wide, without a sudden shrubby edge. Larch canopy opens up gradually either without any or with a *Salix*-dominated shrub layer. Forest grazing impacts the outer 40–100-m-wide belt of the bigger forest patches while smaller forests are grazed through totally.

*Grasslands* are meadow steppes. Ungrazed stands are rich in tall herbs and bushes. However, as most of the grasslands of the study area are moderately or heavily grazed, grass height is low (< 10–20 cm). Grasslands have a closed grass layer, which only opens up on dry, sunny, and rocky slopes. Pebble banks (*sayr*) are often sparsely covered with vegetation (< 20%). In spite of being flooded periodically, pebble banks are dry most of the time. Wet grasslands only occur along water courses; wetter types are tussocky. There is no marsh vegetation in the study area, but there is a bigger river to the west and a salt lake to the east. Limestone outcrops facing south have a sparse vegetation while rock vegetation on north-facing slopes and surrounded by forest is shaded and moist.

### Data collection and analysis

Data collection on traditional ecological knowledge related to folk habitats was based on indoor and outdoor face-to-face interviews, interviews during landscape walks, and participatory fieldwork.

The study was conducted from 2016 to 2018 by a team of orientalists, botanists, and ethnoecologists. The orientalist/Mongolist of the research group is an expert of Mongolian studies including the ethnozoological knowledge of Mongolian herders [37]. He provided cultural insights for the other Hungarian members of the research team that helped reduce or avoid misinterpretations of the data.

In July–August 2016, we visited the area and contacted the families who would be available and willing for our research project in the longer run. Prior, inform consent was asked for following the guidelines of the International Society of Ethnobiology (ISE, 2006). The preliminary list of plant species and habitats recognized and named by locals in the Seruun Gilad area was prepared by conducting structured interviews using colored photos regarding the local flora of the region [45]. Furthermore, photos were taken to show the salient and/or common local plant species and characteristic landscape elements (potential folk habitats).

In June 2017, we visited the same herder families as in 2016. Thirty-nine to 41 pictures of local wild plant species were shown asking them—in a semi-structured way—to name the species and explain the general features and habitat preferences of these species. Additionally, 25+ pictures depicting local habitats were shown to our interviewees in order to elicit local habitat names and the partitioning of the local landscape into folk habitats. During landscape walks, we visited with herders all parts of the study area and we collected additional data on recognized and named plant species and habitat types and made additional documentary pictures for use in future interviews. Additionally, we used 40 plant species pictures for a pile sort exercise and also asked herders to free list main pasture types.

In July 2018, we conducted structured interviews. We asked the locals about the habitat preference of altogether 76 salient local wild plant species. We were able to collect more than 8 independent datasets regarding all the 76 plant species (8 families, i.e., ca. 75% of the families living in the valley). For the habitat preference of a further 64 species, we asked a knowledgeable and experienced herder. Data were collected indoor, but we made outdoor (walking) interviews and participatory fieldwork as well.

Altogether, we interviewed 20 people (15 male and 5 female) from 11 families, 18 of them were full-time herders. The number of informants was limited as landscapes further away from the research site were ecologically different. Interviewees were between 30 and 70 (average 45) years old. Interviews were conducted in Mongolian by GB and ÁA. Interviews ranged from 15 to 118 min. All interviews were digitally recorded (usually both by voice recorder and camera).

First, we transcribed all relevant parts from the interviews. Then, we merged all habitat preference data for each plant species, then grouped all habitat data into main habitat types (defined by the authors, so they do not necessarily reflect local understandings) and described their scientific meaning in English and also provided literal translations. Later, habitats were classified into macro-, meso-, and micro-scale habitats (cf. [22]). Macrohabitats occupy usually large areas and comprise many habitat types (a mosaic), mesohabitats are usually smaller in extension and homogenous and are often dominated by one vegetation type, and microhabitats are embedded in mesohabitats and provide special niches for particular species. We grouped species according to the major folk habitat categories assigned by herders and also into groups of specialist, generalist, and intermediate species based on the specificity or the number of folk habitat types herders attributed the species to. We also provide some information on the most salient indicators herders used to describe and distinguish habitats. Text in italics indicates original quotations and local Mongolian folk names of species and habitats.

## Results

### Folk habitat categories in the Gilad valley

**Fig. 3:**
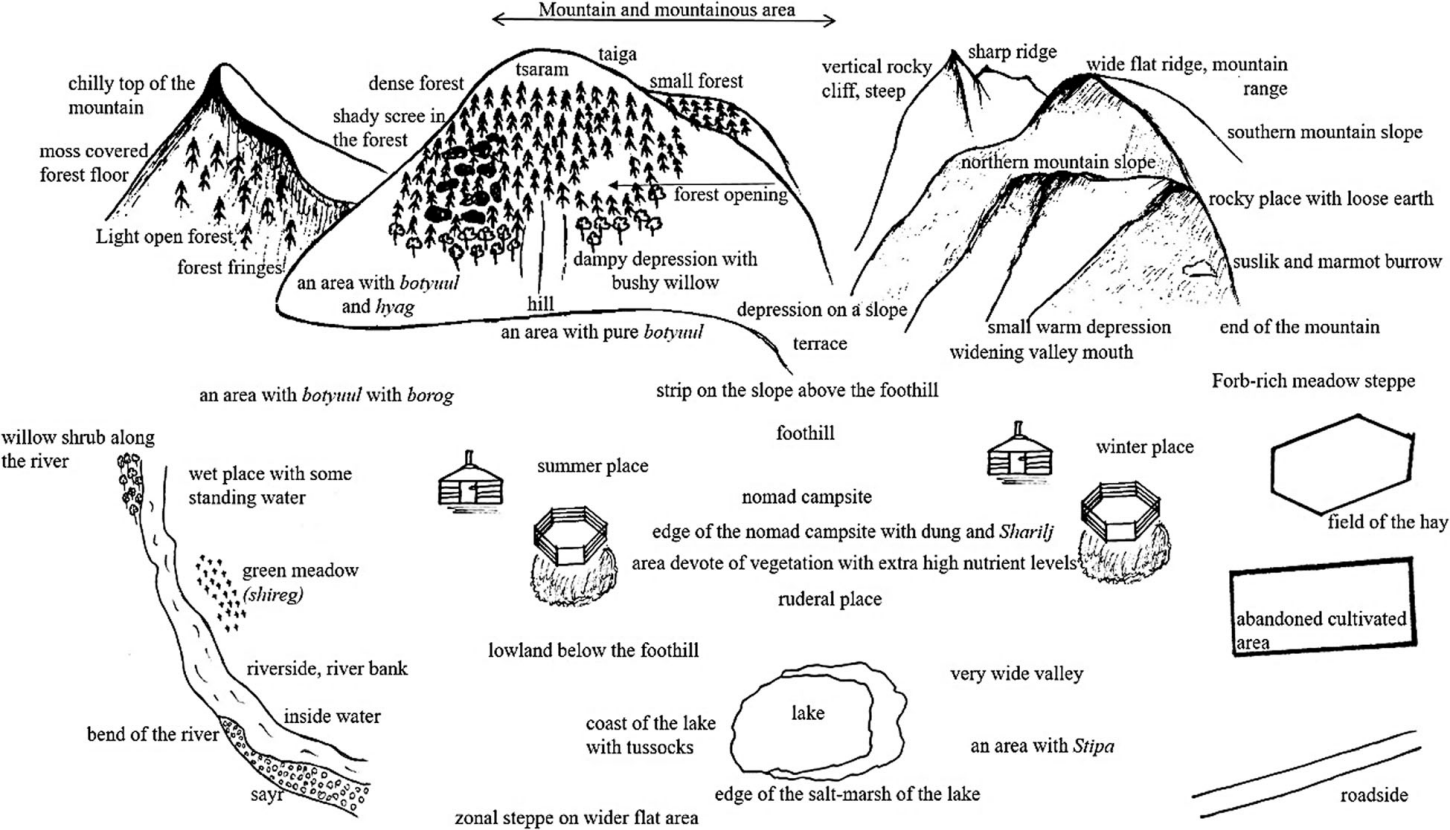
The 59 most salient Mongolian folk habitat categories (Seruun Gilad, Arbulag soum, Khuvsugul province, Northern Mongolia). For Mongolian names and literal translations, see Tables 3, 4, 5, 6, 7, 8, and 9

**Fig. 4:**
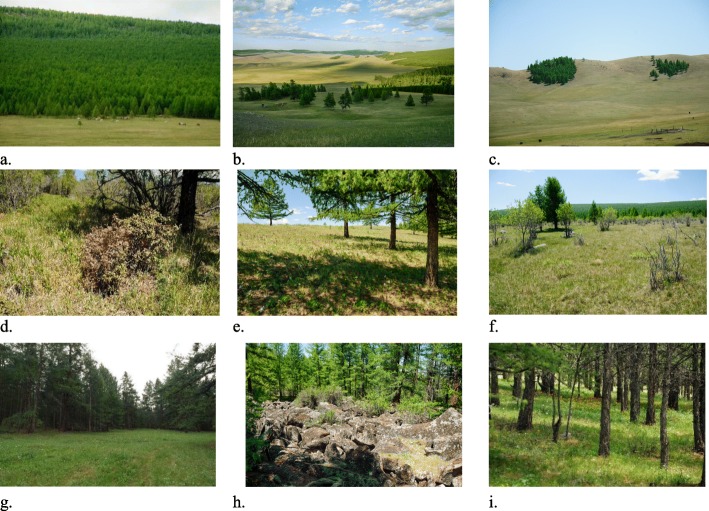
Forest folk habitats. **a** Dense forest. **b** Light forest. **c** Small (orphan) forest. **d** Taiga forest (Rhododendron parvifolium Adams). **e** Chilly strip on the northern side close to the ridge (tsaram). **f** Forest fringe (Salix pseudopentandra (B.Flod) B.Flod.), S. divaricata Pall. **g** Forest opening (also a hayfield). **h** Screes in the forest. **i** In the forest (Trollius asiaticus L.)

**Fig. 5:**
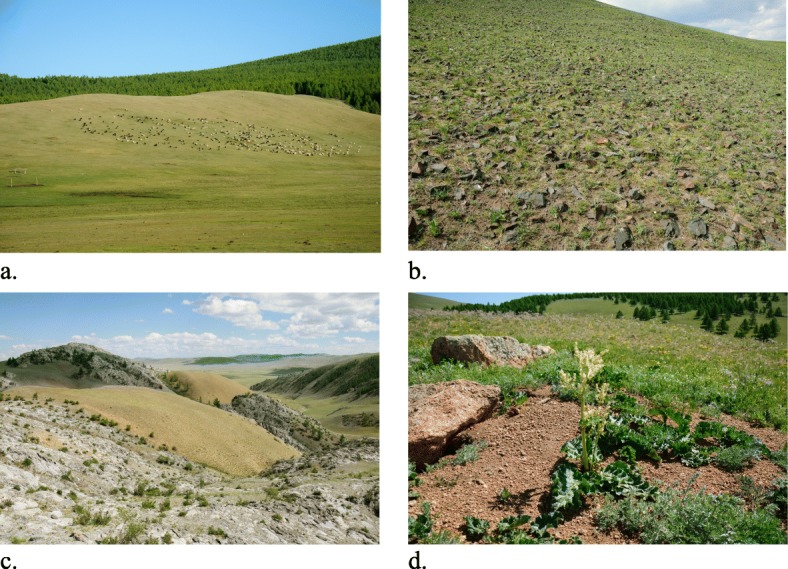
Geomorphology-related folk habitats. **a** Mountain slope with botuul (Festuca lenensis Drob.). **b** Mountain stony slope. **c** Mountain sandy and rocky slopes. **d** Mountain slope with marmot burrows (latter is a microhabitat)

**Fig. 6:**
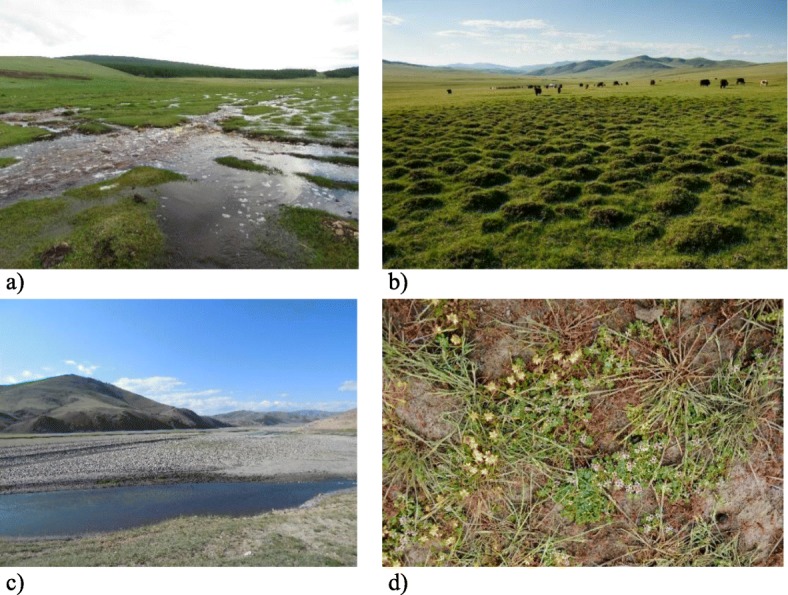
Hydrology-related folk habitats. **a** Green meadow (shireg). **b** Tussocky place. **c** Sayr and river side. **d** Marshland (edge of the lake)

**Fig. 7:**
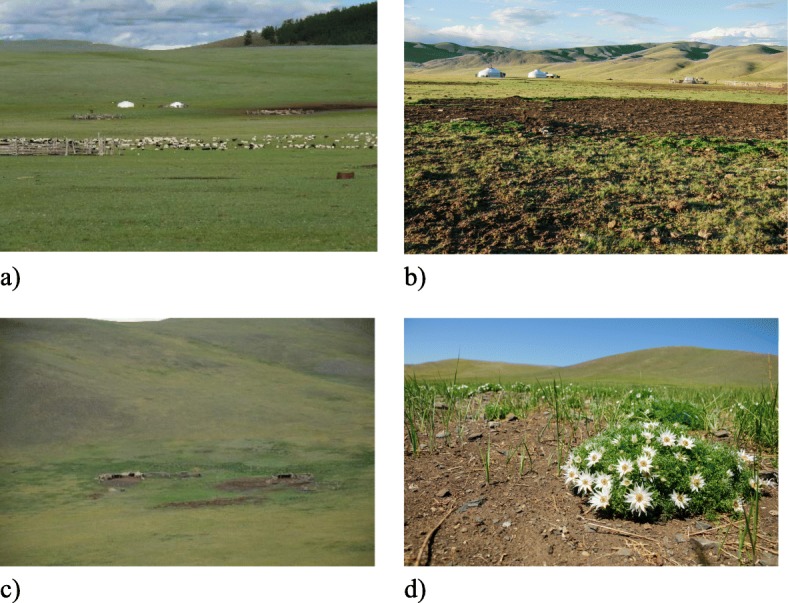
Folk habitats of campsites. **a** “Near the yurt” and “along the fence” at a summer campsite. **b** “Near the dung” and “lifeless earth” at a summer campsite. **c** “Near the shed” and “near the dung” at a winter campsite. **d** “Warm place” and “near the fence” at a winter campsite

Locals in the Gilad valley distinguished altogether at least 88 folk habitat categories (Figs. 3, 4, 5, 6, and 7; Tables 1, 2, 3, 4, 5, 6, 7, 8, and 9). The main categories people partitioned the landscape into were forests, wet areas/lakes/rivers/streams, rocky areas/deep valleys, and dry “normal” pastures in valleys, flat areas, and gentle slopes. The most often mentioned habitat types were as follows: *uuliin enger*, *öwör* (mountain south slope), *uuliin bel* (foothill), *oi dotor*, *modon dotor* (in the forest), *sudag*, *sudgiin zah* (shallow coomb), *buudal gazar* (nomad campsite), *oin zah* (forest fringe), *oin tsol tsoorhoi*, *oin chölöö* (forest opening), *had asgan dund* (between rocks), *goliin zah* (riverside), *tal höndii* (valley), *öwöljöönii gazar* (winter place), *chiigleg gazar* (dampy area), *uuliin ar*, *ar hayaa* (north slope of a mountain), *uul* (mountain), *uuliin zoo* (wide mountain ridge), *taiga* (taiga), *tsaram* (chilly strip on the northern side close to the ridge), *hadarhag gazar* (rocky place), and *jalga* (coomb).

**Table 2:** Macro-scale folk habitat terms (as spoken during the interviews and synonymous names are grouped under the same headings), their scientific meanings, and typical literal translations from the Seruun Gilad, Khuvsugul province, Mongolia. Synonymous Mongolian names are listed in brackets in the second column

Macro-scale habitats	Literal translations	Meaning of the habitat type
тайга, хөвхтэй тайга, хөвдтэй тайга, асга хадтай тайга, тайгархаг ой, тайга сэрүүн газар	Taiga, taiga with litter, taiga with moss, taiga with rocks, taiga forest, chilly taiga	Taiga (dense mossy forest)
уулархаг газар, ойтой уулархаг газар	Mountainous area, mountainous forest area	Mountainous area
тал хээр (тал хөндий)	Plain steppe (plain valley)	Zonal steppe on a wider flat area
говь газар	Gobi place	Gobi (dry steppe and semi-desert)
өвөр газар	Inner place	Inner place (area close to valley bottoms and yurts)
ар газар	North place	Outer place (area further from inner place)
эр газар, эрдүү газар	Wild place	Wild uninhabited place
зуслан газар	Summer place	Summer place (summer pasture area)
өвөлжөө газар (өвөлжөө бууц)	Winter place (öwöljöö buuts)	Winter place (winter pasture area)
сум	Soum	Soum (disturbed habitats of a settlement)

**Table 3:** Forest, forest edge and shrub habitats, and their meaning and literal translations. Synonymous Mongolian names are listed in brackets in the second column

Forests	Literal translations	Meaning of the habitat type
ой, хар модон ой, ой (модон) дотор, уулын арын модон дотор, модон доторх асганы ойролцоо, нураг чулуутай модон дотор, ой доторх бургасан дунд, ойн гүн	Forest, larch forest, in the forest, in the forest of mountain backside, near the rock in the forest, in the forest with scree, between willow in the forest, depths of the forest	Forest (in the forest)
тайгажуу (тайгархаг) газар	Place like taiga	Place like taiga in the forest steppe zone
шигүү ой, өтгөн ой (гахайн шивээ, гөрөөсний шивээ, тургийн шивээ), том ар мод, туж, хөвч	Dense forest, closed forest (*gahain shivee*, *göröösnii shivee*, *turgiin shivee*), tom ar mod, tuj, hövch	Dense forest, closed forest
сийрэг ой (эрээн, цоохор модтой газар, тарлан ой)	Sparse forest (*ereen, tsoohor modtoi gazar, tarlan oi*)	Light open forest
хөгшин ой	Old forest	Forest with old trees
үхмэл ой	Dead forest	Dying forest (old trees without young ones)
нөхөн төлжих чадвартай ой	Renewable capacity forest	Forest that regenerates well
чирэнгэн ой (ширэнгэ ой)	Forest like jungle	Dense young forest (DBH 5–10 cm)
залуу ой	Young forest	Young forest
төгөл (төгцөг, лам чирэнгэ, зураа мод), өнчин зураа, бөөн (хэсэг) мод	Grove *(tögtsög, lam chirenge, zuraa mod*) orphan forest, clump of trees	Small forest (forest patch)
Forest fringes and bush vegetation		
ойн (модны) зах, модны хаяа, ойн хормой, модны тавиу ар хормой, модны хаяан дахь бургастай газар	Edge of the forest, lap of forest, wide lap of forest, area with Willow in the border of forest	Forest fringes
царам, уулын царам, модны (ойн) царам	Alpine (*tsaram*), *tsaram* of mountain, *tsaram* of forest	Grassland strip (including the forest edge) on the chilly, windy northern side close to the top (alpine area around the local tree line)
ойн (модны) цол цоорхой, ойн чөлөө	Gap of forest, forest openings	Forest opening
судаг газар, чийгтэй судаг (судагдуу) газар, явган бургас борогтой судаг газар, өвөр газрын судаг, ойн чийглэг судаг, бургас харганатай судаг дотор	Shallow coomb, dampy shallow coomb, shallow coomb with willow and *borog*, shallow coomb of inner place, dampy shallow coomb of forest, in shallow coomb with willow and pea shrub	Dampy shallow or on northern slopes with bushy pea shrub and willow
чийглэг судгийн зах	Edge of dampy shallow coomb	Edge of dampy shallow coomb on the northern slope
голын захын бургас дагуу, голын ойролцоох бургасан төгөл дунд	Along the willow of riverside, in willow grove near the river	Along willow shrub of the river and stream

**Table 4:** Dry and mesic grassland habitats and their meaning and literal translations (other grassland types are listed in Tables 5, 6, and 8)

Dry and mesic grasslands (including grass-dominated rocky places)	Literal translations	Meaning of the habitat type
уулын нуга	Meadow of the mountain	Forb-rich meadow steppe near the forest on mountain slope
ботуультай газар	An area with *botyuul*	An area with only *botyuul* (dominated by *Festuca*)
ботууль, хиагтай газар	An area with *botyuul* and *hyag*	An area with *botyuul* and *hyag*
ботууль, борогтой газар	An area with *botyuul* and *borog*	An area with *botyuul* and *borog*
боргорхуу (борогдуу) газар, борог-ширэг газар (борог-судаг газар)	Borog place, *borog*-*shireg place* (*borog*-*shallow-coomb place*)	an area with *borog* and *shireg*
хадлангийн газар (талбай), хадлангийн хөцөөн дотор	Hay field (hayland, meadow), into the fence of the hay	Field of hay, into the fence of the hay
ашиглагдахаа больсон тариалангийн талбай	Abandoned area	Abandoned cultivated area

**Table 5:** Wetland habitats and their meaning and literal translations

Wetlands	Literal translations	Meaning of the habitat type
чийглэг газар, чийгэрхэг хөндий (тал), ус шандтай чийглэг газар, ширэгтэй чийглэг газар, хад асгатай чийглэг газар	Dampy place, dampy valley (plain), dampy place with natural spring, dampy place with *shireg*, dampy place with rocks	Dampy place in general, with high groundwater
ширэг газар, голын захын ширэг	Green grass (*shireg*), green grass near riverside	Green grass (*shireg*), closed sward along stream or river
довтой газар, гол дагуух дов тонтуултай газар, чийг эхтэй тонтуултай газар	Tussocky place, tussocky place along river, wet \tussocky place	Meadow with tussocks along streams
хар шороотой газар; усархаг, чийгэрхэг хар хөрстэй газар	Place with black soil, watery and dampy place with black soil	Black soil (with high soil organic matter or humus)
голын (томоохон гол мөрний) зах, голын хажуу, голын эрэг, голын захын чийглэг газар	Edge of the river, riverside, coast of the river, dampy place of edge of the river	Riverside, riverbank
голын булан тохой, цахилдганатай голын тохой	Bend of the river, bend of the river with *swordflag* (*Iris*)	Bend of the river
усархаг газар, борогтой усархаг газар, цахилдганатай усархаг газар	Watery place, watery place with *borog*, watery place with *swordflag*	Wet place with some standing water
нуур тойромтой газар, нуур цөөрөм тогтдог газар, тогтоол устай газар, ус тогтсон газар	Place with lake, place with ponds, place with dead water or backwater	Lake, depression with standing water
мөнх булаг шандны хажуу	Side of the natural spring	Side of the natural spring
усан дотор	In water	Water (as habitat)
нуурын эрэг, нуурын зах (хөвөө) тонтуултай газар,	Coast of the lake, edge of the lake with tussocks	Coast of the lake with tussokcs
хужир мараатай нуурын зах, хужиртай мараархаг (мараалаг) газар	Edge of the lake with salt, marshland with salt	Edge of the salt-marsh of the lake

**Table 6:** Rocky and stony habitats and their meaning and literal translations

Rocky and stony area	Literal translations	Meaning of the habitat type
хясаа, хясаа хадтай уулархаг газар, ганга газар (уулын эгц /хэц/ газар), хавцал, асга хадтай эгц хавцал, модтой хавцал газар	Vertical cliff, mountainous area with vertical cliff, steep (steep place), canyon, canyon with cliffs, canyon with tree	Vertical rocky cliff, steep mountain slope
хадлаг газар, хадны завсар хооронд (дотор), сул хөрс бүхий хадтай газар, хүрэн хадтай газар; элс хөх хайртай, өвс муутай, цахир хадтай газар	Rocky place, between the rock, rocky place with loose soil, place with brown rock, place with sand, blue stone, less grass and flint rock	Rocky place (rock sticking out of the ground) in general, including sunny scree
хадны нөмөр, уул хадны ёроол	Leeward of rocks, bottom of the mountain and rock	Bottom of the rocks
хад асган дунд (дотор), нураг асгатай газар, хад асгатай бартаат газар	Between rock cliff, place with scree, obstacle place with rock cliff	Shady scree in the forest on northern slope
хайдам /нүцгэн сарьдаг/ газар	Rock mountain (haidam)	Large treeless rock outcrop
чулуурхаг газар; уулын хадархаг, чулуурхаг, элсэрхэг газар; хөх чулуу, хөх нуранги шороотой газар	Stony place; rocky, stony and sandy place; place with blue stone and ground	Stony place in general
голын сайр, сайран дээр, гол хоорондын сайр, голын хуурай сайр (ус ширгэсэн газар)	Sayr of river, on the sayr, sayr between rivers, dry sayr of river	Sayr (regularly flooded place with rounded pebbles along and among river beds)
дайргатай газар; элс дайрга, алтан харганатай газар	Place with unrounded stone (dairga), place with sand, dairga and pea shrub	Rocky and sandy surface in and along streams at the mouth of narrow valleys with less rounded stones (difficult to cross)
халуун элсэрхэг, чулуурхаг, хадархаг газар; цагаан ботууль, хялганатай, элсэрхэг, хадлаг, хатуу газар; уулын энгэрийн халуун газар	Stony and rocky hot sandy place; sandy, rocky place with white fescue and feather-grass, hot place of mountain south slope	Hot place (sandy, stony and rocky)
сул хөрстэй газар (нурмаг шороотой газар), элсэрхэг сул хөрстэй газар,	Place with loose soil, loose soil with sand	Rocky place with loose soil

**Table 7:** Degraded habitats and their meaning and literal translations

Disturbed areas	Literal translations	Meaning of the habitat type
малын (хуучин) буудал газар, малын өтөг бууц	(Old) nomad campsites, dung of livestock	Nomad campsites in general
хөл газар, үхэл хөрстэй хөл газар, гэрийн ойролцоо	Ruderal (foot) place, foot place with lifeless soil, near the yurts (ger)	Ruderal place (heavily trampled by humans and livestock)
хөрс нь гэмтсэн (эвдэрсэн) газар	An area with damaged (eroded) soil	An area with soil destroyed by livestock and humans
үхмэл газар, хөрс муутай үхмэл газар, үхмэл буудал газар	Dead place, dead place with low nutrient soil, dead nomad campsite	Area devode of vegetation with extra high nutrient levels (caused by trampling and resting by livestock)
сумын төвийн хашаан дотор	In the yard of center of the soum	Yard in a settlement

**Table 8:** Geomorphologically defined habitats and their meaning and literal translations

Mountainous area, mountain slopes	Literal translations	Meaning of the habitat type
уул	Mountain	Mountain
уул толгод	Hill	Hill
уулын энгэр (өвөр), хад асгатай уулын энгэр, хад багатай уулын энгэр, сул хөрстэй уулын энгэр, хадлаг уул толгодын энгэр, чулуурхаг уулын энгэр, талархаг энгэр, өвөлжөөний бууцтай энгэр газар, уулын налуу (ташуу, хажуу) газар	Lapel (front side) of mountain, lapel of rocky mountain, lapel of mountain having less rock, lapel of mountain with loose soil, lapel of rocky mountain hill, lapel of stony mountain, wide lapel, lapel with nomad campsite of winter place, mountain slope	Southern mountain slope, sunny slope
уулын ар (газар)	North slope of mountain	Northern mountain slope
уулын хормой (хаяа, ар хаяа), тайгадуу өндөр уулын хормой,	North slope (lap) of mountain, slope of high mountain like taiga	Strip on the slope above the foothill, lower part of the northern mountain slope
уулын (өвөр) бэл, уулын нөмөр энгэр бэл, нураг хөрстэй хадархаг уулын бэл	Foothill, (inner-foothill), leeward-foothill, foothill of rocky mountain with loose soil	Foothill
бэлэрхүү нам дор газар, нам дор газар	Lowland-like foothill, lowland	Lowland below the foothill
уулын хамар, уулын хадархаг үзүүр, уулын шувтарга үзүүр, уулын ар хошуу	End (nose) of mountain, rocky end of mountain, final end of mountain, back end of mountain	End of the mountain
даваан дээр	On the pass	Pass
дэвсэг (хад асгагүй газар), хуурай дэнж, зэрлэг дэнж газар, цагаан дэнж газар	Terrace (no rock place), dry hilltop, wild hilltop, white hilltop	Terrace
уулын хяр, уулын ирмэг газар, ус багатай хяр газар	Mountain crest, border place of mountain, mountain crest with less water	Sharp ridge
уулын зоо, уулын нуруу, ар зоо	Mountain range, back (spine) of mountain	Wide flat ridge, mountain range
уулын таг сэрүүн газар, модны таг сэрүүн газар, уулын дээд хэсэг, өндөрлөг сэрүүн газар	Alpine and cool place of mountain, cool place of forest, upperside of mountain, highland and cool place	Chilly top of the mountain (parallel to tsaram)
Valley and depression		
тал хөндий, тал (талархаг) газар, талархуу тэгш газар, хатуу хөрстэй тал газар	Valley, plain (like plain), flat area like plain, plain with hard soil	Very wide valley bottom
гуу жалга, модны ойролцоох гуу жалга (модны суга жалга, уулын суга жалга), чийглэг жалга, өвөлжөөний чийгтэй жалга, гүн жалга, ховил жалга, жалга дотор, жалга гууны ам	Coomb, coomb near the forest (armpit-coomb of the forest or mountain), dampy coomb, dampy coomb of winter place, depth coomb, groove-coomb, in the coomb, mouth of coomb	Coomb on the mountain slope
жалгын зах	Edge of coomb	Edge of coomb on a slope
сүүдэрлэг аглаг (ар) газар, сүүдэрлэг, хөр их хураасан газар, ойн сүүдэрхэг газар	Shady wild place, shady place with snow accumulation, shady place of forest	Shady place with snow accumulation
уулын ам	Mouth of mountain	Widening valley mouth
дулаан газар, хонхор дулаан газар, нөмөр дулаан газар, өвөлжөөний нөмөр дулаан газар	Warm place, hollow-warm place, leeward warm place, warm place of winter place	Small warm depression often on southern slope with less wind and snow

**Table 9:** Micro-habitats and their meaning and literal translations

Micro-habitats	Literal translations	Meaning of the habitat type
хулгана зурамны нүх, тарваганы дошон дээр, тарваганы доштой уулын энгэр, уулын доширхог газар	Mouse and suslik burrow, on the marmot burrow, mountain slope with marmot burrow, burrow in mountain	Bare and stony surface around mouse, suslik and marmot burrows
хадан дээр	On the rock	On the rock
дов сондуулын толгой дээр	On the top of the tussock	On the top of the tussock
хөвх ихтэй газар, хөвд ихтэй газар, хөвдөрхөг - чийгэрхэг ар газар	Place with litter (forest floor), mossy area, dampy area with moss	Moss-covered forest floor with decomposing litter
буудлын зах, буудлын захын шарилжтай газар, шавхайны бууцны зах, хөрзөн өтөгний хажуу, малын хашаа хорооны хажуу	Edge of the nomad campsite, edge of the nomad campsite having *sharilj*, edge of the manure, side of dried dung, near the fence	Edge of nomad campsite with dung and *sharilj*
хогон дээр, хогны хажуугаар	On the waste, close to waste	On and near rubbish dump roadside
замын хажуу	Side of the road	Roadside
хөрс сайтай газар	Fertile soil place	Naturally fertile soil in general
хөрсжиж байгаа газар, (эдгэрч буй газар) хуучин эдгэрсэн буудал	Recovering soil, old recovered nomad camp sites	Formerly denuded area (by livestock or humans) with regenerating vegetation (abandoned yurt and pensites)

Below, we list and describe all folk habitats grouped into the following main categories: macro-scale habitats, meso-scale habitats, and micro-scale habitats.

### Macro-scale habitats

Macro-scale habitats refer to larger areas with a mosaic of several/many habitat types. Herders distinguished some main pasture complexes at the supra-local level: mountainous pastures of the Khangai region (to where the study area belongs), steppe pastures of wide flat valleys and lowlands (to the south with none or much less forest cover), taiga (densely wooded pastures to the north in the forest zone), and Gobi (desert pastures far to the south) (Table 2). In the case of some species, they referred to these far away areas when asked about habitat preference (Gobi: *Allium mongolicum* Turcz. ex Regel, *Ulmus pumila* L.; taiga: *Rhododendron parvifolium* Adams, *Juniperus sibirica* Burgsd.). They also mentioned areas *where there is no permafrost* and settled area (cities) as macro-habitats.

Herders partitioned the local landscape into “south” and “north” (outer) parts. South areas are closer or around the summer and winter camps and often get more manure. They said: *Agi* (*Artemisia frigida* Willd., an indicator of overgrazing) *grows in south areas*. In contrast, north (outer) areas are less disturbed, non-manured and less used by people (*they are more wild*). South areas are better for small ruminants while outer areas are better for large livestock.

Mongolians have seasonal pastures. Winter places (pastures utilized between October and May) were often mentioned as a macro-scale habitat. Winter places have some common features compared to summer (or spring and autumn) places, as they are located in less windy areas and close to warmer sunny slopes with less snow. Winter places tend to have more rugged geomorphology with deep rocky valleys. Winter campsites (the center of the winter places, with nutritious grasses) is a meso-scale habitat (see below). Herders said that *the grass is keep growing* (regrowing in the same season after being grazed) *on summer pastures* (*every day the grass grows*) but not on winter pastures. In winter places, the goal is to *fill the stomach of the livestock*, as the grass is not nutritious, because *nutrients went down into the roots*.

Cities (as a complex of weedy, disturbed habitats) were mentioned as a habitat for some species like *Chenopodium album* L. and *Plantago depressa* Schlecht.

In the case of some species (like *Potentilla fruticosa* L., *Aster alpinus* L., *Gentiana decumbens* L.fill.), herders found it difficult to assign them to specific habitats because of their widespread distribution. They said these species grow “everywhere,” which usually meant that they do not have a strong habitat preference (cf. generalist species), they can grow in many not-too-wet, not-too-rocky, and non-forested habitats. In other cases (like *Achnatherum splendens* (Trin.) Nevski, *Allium altaicum* Pall., *Iris lactea* Pall.), they also said “everywhere” but indicating that the species grows in most valleys and slopes in this region of Mongolia.

### Meso-scale habitats

#### Forests, shrub vegetation, and forest fringes

Forests are salient habitats in this landscape. Most forests are located on the northern side of the mountains. Locals distinguished 13 forest types based on the structure (density), age, and health status of the forest (Table 3). They also distinguished coniferous forests (*shilmüüst mod*, referring to *Larix* leaf) from leafy forests (*navchit mod*, leaved trees). However, the latter category did not occur in the study area. Herders also distinguished hard trees (*Larix sibirica* Ledeb., *Betula* sp., *Populus* spp.) and soft trees (*Pinus sibirica* Du Tour, *Picea obovata* Ledeb.). Soft trees do not grow alone, they always form large patches—herders said.

The area of forests usually does not increase under this cold continental climate. Some herders said that present forest patches came to exist when the world was created, similarly to rocks. *Forests were scattered in the landscape at that time, and this is what we have now*. Herders argued that the spatial pattern of forest patches has been stable on longer term (centuries). The average diameter of trees does change (grow), but the place of the forest would never change, *not even after an earthquake*—they said. Tree growth is very slow in this area, trees live long, and herders separated forests that have a good future (renewable forest) or bad future (dying forest). Herders said that some forests have a rejuvenating ability (*if you cut the trees, they will regrow*); in other cases, the forest is “dead” (i.e., will not regenerate if cut because there are no young trees among the old ones). The small forest patches were called orphan forests (*önchin zuraa*, *zuraa mod*) and protected from extensive wood cutting. Forests are open and sparse in windy places.

Locals do not usually cut trees near the forest edge and in small forests neither trees with specific characteristic shape, because they argue the cutter would be threatened by a lightning strike or be injured. They cut their firewood (only dead wood) from the forest interior in autumn. They argued: *Everything in nature revives/regrows when spring starts, and becomes sleepy when autumn starts. Everything is connected in nature. Therefore we cut our firewood in autumn*.

*Pastures in or near the forest* are important for livestock. Herders distinguished four pasture types in the forest: (1) *patch with willow*, (2) *borog* (*Carex* spp.) *along the stream or water*, (3) *leafy plants*, and (4) *hyag* (*Agropyron cristatum* (L.) Beauv.). In droughts, herders will drive their livestock into the forest and some areas close to the forest, because there is water there (the area is dampy, they said, *roots of trees keep a lot of water,* ca. *300 litres water* per tree). In the winter, *forests are warm and they are fine for the livestock*. They added that in severe and heavy rain, they will graze their livestock in the forest. The forest grasses are fresh. Herders argued that pastures among the trees are worse (are of lower nutritional quality) than the pastures on dry mountain slopes. Sheep sometimes goes (without the supervision of the herder) deep into the forest (further in than the forest fringe zone) to graze on *darsh* (*Vicia amoena* Fisch.). Herders said: the deep forest is dangerous for the livestock because of carnivores and *I drive the sheep out from the forest before sunset because of wolves*. Horses may go even deeper into some forests as they are free ranging. Livestock grazes less in the forest in summer and more in autumn. Herders added that it is good to have the noon rest in the forest. They also added that *if there is snow before the grass dies, and it remains green during the fall, grass in the forest will stay green and keeps under the snow until spring. It means that nature is making a plant silage itself. This green grass is beneficial for the livestock*.

There are only few shrub-dominated places in the study area, willows (*Salix pseudopentandra* (B.Flod.) B.Flod., *S. divaricata* Pall.) being the most common shrub species. Willows grow along forest fringes and in wet meadows (red willow (*Salix turanica* Nas.) grows in *borog* place, see below). Willow shrub is a key pasture habitat in spring and summer, herders said: *willow has a bitter taste* (=good), *I drive them into the willow shrub in the morning. Willow shrub is a natural oasis, it is a crucial pasture in droughts, it is good against parasites.* Grasses growing among willows are “hotter grasses” (see below), they are greener, because the habitat is more dampy and shady. Most shrub species are grazed by the livestock; thus, they can often only survive among rocks out of reach of animals.

Herders distinguished four forest fringe types: (1) with willow, (2) with *pea shrub* (*Caragana jubata* (Pall.) Poir.), (3) with *hyag* (*Agropyron cristatum* (L.) Beauv.), and (4) with rocks. Most forest fringes are important pastures, especially those on the *backside* (northern slopes). Forest fringes turn yellow later, only in late autumn. These habitats are full of *hyag* and leafy species (like *Bromopsis pumpelliana* (Scribn.) Holub., *Agropyron cristatum* (L.) Beauv., *Bistorta alopecuroides* (Turcz. ex Meissn.) Kom). Herders said: *leafy plants, hot plants* (like *dagsh: Oxytropis pseudoglandulosa* Gontsch. ex Grub.) *grow close to the forests, it is a good pasture, sheep has a good time there*.

#### Dry and mesic grasslands

There are many different types of grasslands in the Gilad area according to herders, for example, the grasslands on sunny slopes, foothills, plains, on the northern sides, in forest openings and along forest fringes, on rocks, and in the hay fields of the winter places (Table 4). *Grass is the green gold*—herders argued.

Herders said that *grass is denser and taller* on the northern slopes because the site is *more shady and has a better water supply*. The grass is more palatable but less nutritious, and the *animals are cold* there in the winter. These slopes have a lot of *borog* (here *Carex pediformis* C.A.Mey., *Poa pratensis* L., *Kobresia* spp.) – herders said. The uppermost part of the northern slopes is a cool windy habitat with some specialist species (*this plant* (*Dryas oxyodonta* Juz.) *does not grow at lower elevation*). The grass is *thin* and (structurally) *fine*, *livestock likes to be there*.

Southern slopes are *sunny, grass is less dense, sparse*, and *always drying out*, but of *higher forage quality*. All herders agreed on these. The soil on the southern slope is relatively dry and dries up quickly. Roots of the plants are sparse; the land is often *grassless*. Sheep and goats graze a lot there in spring because the *roots of the plants unfreeze early in the spring*. Sheep and goats (also called *thin animals*, *nariin mal*) like grazing on southern places. They also added that *on dry mountain, slopes there is no hyag* (*Agropyron cristatum* (L.) Beauv.), *leafy plants, and sharilj* (*Artemisia macrocephala* Jacq. ex Bess. and some other ruderal *Artemisia* species). Herders said that they must graze animals in these inner pastures, especially early morning, in cool spring and autumn and in cold winter (*you have to start the grazing route here in the morning, livestock will give a lot of milk, it will fatten well*). They said what Mongolians often recall that: *You do not feel cold, if you follow the sun, and you do not feel hungry if you follow the animals.*

In free listings of pasture types, herders usually answered with a list of dominant folk taxa: *botyuul* (*Festuca lenensis* Drob.), *borog* (*Carex duriuscula* C.A.Mey. and other species), *hyag* (*Agropyron cristatum* (L.) Beauv.), *ders* (*Achnatherum splendens* (Trin.) Nevski), *shivee* (*Stipa* spp.), *sharilj* (*Artemisia tanacetifolia* L., and other species), and *luul* (*Chenopodium* spp.). In other cases when we asked about the main grassland types, they listed (1) *shireg* (grasslands near the stream, in valleys, (2) *botyuul* (*Festuca lenensis* Drob. on the slopes), (3) leafy-hot grasses (around forests on the slopes, with *hyag* and *botyuul*), and (4) *hyag* at winter campsites.

Herders always argued that *botyuul* is *very good for the livestock*, *animals like eating it*, and *fatten quickly on it,* adding that *botyuul is the best grass what livestock can find to graze upon in Mongolia*. When 40 plant species were pile-sorted by a herder, after 40 min of sorting species into trees, flowering plants, leafy plants, hot plants, summer forages etc., he lifted the picture of *botyuul*, and said: *and this is THE botyuul*!—which did not belong to any of the piled groups. Herders listed four types of *botyuul*: gold, red, white (or thick), and *yanzagan* (fawn). They also said that *botyuul* dominates in three pasture types: *botyuul* with *borog* (where we found *Festuca lenensis* Drob., *Carex* sp., *Agrostis trinii* Turcz., *Koeleria cristata* (L.) Pers.), *botyuul* with *hyag* (with *Festuca lenensis* Drob., *Agropyron cristatum* (L.) Beauv., *Poa palustris* L., *Bromopsis pumpelliana* (Scribn.) Holub.), and pure *botyuul* (*Festuca lenensis* Drob.). *Borog* is a complex term. *Borog* includes *Kobresia* spp. and *Carex duriuscula* C.A.Mey. and other similar Monocotyledons (i.e., short and dry grasses growing on gently sunny slopes and plain areas), but the word *borog* was also used for dried *shireg* (dried wet meadows, see below).

There are only few habitat types where herders make hay for the winter (and they make only a little compared to the size of the herds). The most important hay meadows are in or near the winter campsites (but also in forest openings and in some drier valleys). Hay meadows are often fenced from livestock, are mown in August, and are dominated by *hyag* (*Agropyron cristatum* (L.) Beauv.). *Hyag* is a hot grass (meaning nutritious). *Hyag* has no flower—herders argued—but it has a *head*. It grows at winter places, in forest fringes and in *shireg*.

#### Wet habitats

Though the area has a continental forest steppe climate, there are many places in the landscape where water comes near or flows on the surface and accumulates in lakes and thus gives rise for specific habitat types, for example, *shireg*, “along streams,” lake, spring, tussocky area, *borog* place, riverside, damp area, marshland, or “bend of the river with *Iris lactea* Pall.” (Table 5).

*Shireg* (green meadow) is one of the most important of these. It is a lawn, a dense sward, which is very damp, and *the bare soil can not be seen*. Its dominant species is *Carex duriuscula* C.A.Mey. (N.B. the official Mongolian name of *C. duriuscula* is *shireg ulalj*). However, locally, *shireg* has two names: *shireg* when it is growing (green grass—*zuleg*) and *borog* after it has dried. Herders argued that *in shireg animals do not fatten, they fatten on dry slopes with botyuul. Cattle grazes in borog in winter*. Greener meadows in a small flat valley are referred to as *borog shireg* or *borog sudag*. *Borog shireg* (with *Festuca lenensis* Drob., *Carex duriuscula* C.A.Mey., *Gentiana squarrosa* Ledeb., *Bistorta vivipara* (L.) S.F.Gray) is found on slopes close to *shireg-*dominated valleys, while *borog sudag* is found along shallow coombs on hillsides (*Festuca lenensis* Drob., *Carex pediformis* C.A.Mey., *Poa* sp., *Kobresia* spp.).

A salient habitat is the tussocky grassland dominated by *Carex duriuscula* C.A.Mey., *Bistorta vivipara* (L.) S.F.Gray, *Cirsium esculentum* (Siev.) C.A.Mey., and *Primula farinosa* L. Herders argued that tussocks grow in height (they are higher than the surrounding surfaces). *They grow where there is water*. *Nature created them, ice, water and drought*. The top of the tussocks is *an important first pasture in spring when the sun melts the snow*. They also observed that *the heads of the tussocks have turned black recently* and they argued that this is *because underground water is decreasing.*

A specific habitat is found around springs with salty water. Similar salty habitats are found around bigger lakes (towards Murun). Here, *the salt grows out from ground.*

#### Rocky, barren habitats

Eight rock habitat types were distinguished by herders based on the size of the stones and rocks, pattern and mobility of these and the vegetation between them, and the location of the habitat in the landscape (close to rivers or surrounded by forests) (Table 6).

They argued that sparsely vegetated rocky slopes are better quality pastures than the dense *shireg* areas along streams. Rock grasslands are often grazed intensively at winter pasture places. *Overgrazing loosens the soil surface and causes erosion*—herders said. Goats especially like grazing on rocky and stony places.

They also separated habitats with stones that are fixed in the ground (like mountain rocky slope, screes in the forest) and stony habitats that have stones lying on the surface (and thus can be moved away easily) (mountain stony slope). One herder listed four types of rocky habitats: (1) *place with tsahir rock* (blue soil) *with less-palatable* (*borog*) *grass, a site inappropriate for livestock grazing*; (2) *place with white stones, black rocks, or red rocks, where palatable grasses grow and thus are good for livestock grazing*; (3) *place with brown rock with Salix* spp., *Cotoneaster melanocarpus* Fisch. ex Blytt., *Urtica angustifolia* Fisch. ex Hornem.; and (4) *place with bad soil, loosened soil, and red sand, such places can easily be degraded by livestock*.

Local rivers are fast flowing and often have floods. Herders distinguished habitats with a high density of stones: *sayr* has pebbles while *dairga* has unrounded stones and is difficult to cross. Both have small vegetation cover.

*Screes* are formed on steeper slopes and may provide refuges for less grazing-tolerant species. Some screes are surrounded by forest and have a damp microclimate. Herders distinguished these habitats as they provide important wild fruits (e.g., *Ribes altissimum* Turcz. ex Pojark., *Rosa acicularis* Lindl.).

#### Degraded habitats

Areas intensively used by humans and livestock were regarded as degraded. These habitas have short grass or are totally bare, grassless. Nomad campsites have a diverse set of disturbed, weedy habitats: animal pens, dung covered areas, weedy areas with *sharilj*, and strips along fences (Table 7). Herders said that rubbish grasses (weeds) like *sharilj* and *luul* (*Chenopodium* spp.) grow on these ruderal (rubbish/debris), *overfed* places, where *the soil is erroneous*. They said that these places provide the first-grown forage in spring. Herders said that on denser pastures, the roots of pasture plants *rule the earth*; thus, *sharilj can not grow there, it has no chance at all, the vegetation is saturated*, so there are no pasture weeds on the pastures further from the summer and winter campsites.

Herders used the expression *lifeless soil* (*ühmel h*ö*rs*) for areas near the yurt at nomad camps sites or on abandoned yurt and pen places where *the soil is “soiling back.”*

#### Geomorphologically defined habitats: slopes and valleys

Herders often referred to geomorphological features when describing habitats of wild plant species: for example, on the southern or northern slopes, on foothills, or on flatter or sharper ridges (Table 8). They argued that valleys have wetter soil because there is more snow there which melts later. Often, geomorphology-based names were combined with other meso-habitat names (e.g., shallow coomb on the mountain northern side, foothill with rock and loose soil).

### Microhabitats

Micro-scale habitats are usually formed at a finer spatial scale than meso-scale habitats. However, the separation is not sharp. Typical microhabitats were the marmot burrows that had disturbed vegetation with species with higher nutrient needs (cf. the manuring effect of marmots and the effect of the loosened soil) (Table 9). *Borog* is growing “on the top of the tussocks,” while *Atragene sibirica* L. “creeps on the tree.” Mossy forest floors are distinguished as a habitat for *Ledum palustre* L., *Vaccinium* spp., *Rhododendron parvifolium* Adams, and *Juniperus pseudosabina* Fisch. et Mey., while weeds accummulate around manure and trash heaps, along fences and roadsides.

### Key dimensions of the local landscape partitioning

Herders distinguished habitats using diverse features. These features could be grouped into some major dimensions (sensu [19, 22]), like geomorphological features (e.g., slope, ridge, coomb), hydrological features (river, lake, wet meadow, marshland), edaphic (bedrock, soil) features (rocks, screes, *sayr*), topography (lowland steppes, high mountain treeless areas), human and animal disturbance (*lifeless* earth, nomad campsites, marmot burrows), dominant plant species (*Larix* forest, *botyuul*-dominated grasslands, *shireg*, *borog*, mossy forest floor), vegetation structure and physiognomy (forest edge, sparse forest, tussock grassland), land use (pastures, hay meadow), and succession (young forest, regenerating grassland).

### Salient plant species of the local landscape partitioning

#### Habitat of one species is given by mentioning another species

In several cases, herders defined the habitat of a species by refering to another species that often co-occurs with that—usually habitat specialist—species (Table 10). *Salix* spp. (mostly *S. turanica* Nas.), *Larix sibirica* Ledeb., and *borog* (small growing *Carex* spp.) were most often used for this purpose.

**Table 10:** Species where their habitat was determined by mentioning its co-occurrance with another species

Species name	… Occurs where species X grows
*Ribes nigrum* L.	*Rhododendron parvifolium* Adams, *Ledum palustre* L.
*Betula fruticosa* Pall.	*Salix* sp., *Allium senescens* L., *Rumex acetosa* L., *borog* (*Kobresia* sp.)
*Carum carvi* L.	*borog* (*Carex coriophora* Fisch. et Mey.)
*Salix turanica* Nas.	*borog* (*Carex pediformis* C.A.Mey.)
*Rhododendron parvifolium* Adams	*Salix* sp. (mostly *S. turanica* Nas.)
*Allium senencens* L.	*Salix* sp. (mostly *S. turanica* Nas.)
*Galium verum* L.	*Iris lactea* Pall.
*Hordeum brevisubulatum* (Trin.) Link	*shireg* (*Carex duriuscula* C.A.Mey.)
*Vaccinium uliginosum* L.	Moss
*Ledum palustre* L.	Moss
*Juniperus pseudosabina* Fisch. et Mey.	*Salix* sp., *Caragana* (*C. jubata* (Pall.) Poir.)
*Juniperus sibirica* Burgsd.	*Salix* sp., *Caragana* (*C. jubata* (Pall.) Poir.)
*Salix* sp. *(tsagaan burgas)*	*Stipa* spp. (*S. glareosa* P.Smirn. and *S. krylovii* Roshev.)
*Gentiana macrophylla* Pall.	*Salix* sp. (mostly *S. turanica* Nas.)
*Atragene sibirica* L.	*Salix* sp. (mostly *Salix pseudopentandra* (B.Flod.) B. Flod and *S. divaricata* Pall.), *Larix sibirica* Ledeb., *borog* (*Carex melanantha* C.A.Mey.)
*Betula* sp. (*hus*)	*Larix sibirica* Ledeb.
Хуслуур (kind of *Betula*)	*Ulmus* sp. (*U. pumila* L.), *Larix sibirica* Ledeb.

#### Habitat specialist and habitat generalist species

We collected habitat preference data for 76 wild plant species (Table 11). Some species occured almost everywhere, and some others had a single specific habitat. Herders often came up with their answers iteratively as many species occured in several habitat types. Typical generalist species in this landscape were according to herders, for example, *Potentilla fruticosa* L., *Pulsatilla turczaninovii* Kryl. et Serg., *Rhododendron parvifolium* Adams, *Allium altaicum* Pall., *Rheum undulatum* L., and *Artemisia macrocephala* Jacq. ex Bess., while habitat specialists were, for example, *Papaver nudicaule* L., *Aconitum barbatum* Pers, *Atragene sibirica* L., *Glaux maritima* L., *Primula farinosa* L., and *Leptopyrum fumarioides* (L.) Reichenb*.*

**Table 11:** Major folk habitat categories and plant species attributed to these major categories by local herders and grouping of these species into specialist (occur only in one or two habitat types according to herders), generalist (occur in many different habitat types or in macro-habitats), and intermediate categories based on the specificity or the number of folk habitat types herders attributed the species to

Main folk habitat categories	Generalist species	Specialist species	Intermediate species
Taiga forest	*Rhododendron parvifolium* Adams	*Tsös övs-Thalictrum* sp.?	*Vaccinium uliginosum* L., *Salix glauca* L., *Gentiana algida* Pall., *Cypripedium guttatum* Sw., *Juniperus sibirica* Burgsd.
In the forest	*Potentilla fruticosa* L.	*Atragene sibirica* L., *Aconitum barbatum* Pers.	*Gentiana macrophylla* Pall., *Cypripedium guttatum* Sw., *Betula fruticosa*, *Trollius asiaticus* L., *Rosa acicularis* Lindl., *Chamaenerion angustifolium* (L.) Scop., *Dianthus superbus* L.
Forest fringes	*Pulsatilla turczaninovii* Kryl. et Serg., *Potentilla fruticosa* L.		*Festuca lenensis* Drob., *Salix turanica* Nas., *Salix pseudopentandra* (B.Flod.) B.Flod., *Salix glauca* L., *Rumex acetosa* L., *Gentiana macrophylla* Pall., *Pulsatilla flavescens* (Zucc.) Juz., *Trollius asiaticus* L., *Betula fruticosa* Pall., *Bistorta alopecuroides* (Turcz. ex Meissn.) Kom., *Rosa acicularis* Lindl., *Sanguisorba officinalis* L., *Gentiana algida* Pall.
Forest openings	*Ledum palustre* L.		*Dianthus superbus* L., *Dianthus versicolor* Fisch. ex Link., *Parnassia palustris* L., *Rosa acicularis* Lindl.
Tsaram (alpine zone, above the tree line)	*Potentilla fruticosa* L., *Rhododendron parvifolium* Adams, *Ledum palustre* L.	*Pulsatilla flavescens* (Zucc.) Juz.	*Achillea asiatica* Serg., *Allium senescens* L., *Chamaenerion angustifolium* (L.) Scop., *Gentiana macrophylla* Pall., *Dianthus superbus* L., *Caragana jubata* (Pall,) Poir., *Trollius asiaticus* L.
Between rocks in the forest	*Potentilla fruticosa* L., *Allium altaicum* Pall., *Rosa acicularis* Lindl.		*Saussurea involucrata* (Kar. et Kir) Sch.Bip., *S. dorogostaiskii* Palib., *Vaccinium uliginosum* L., *Chamaenerion angustifolium* (L.) Scop., *Ulaalzgana - Ribes rubrum* L.?, *Caragana jubata* (Pall.) Poir., *Rhodiola rosea* L., *Ribes altissimum* Turcz. ex Pojark.
Mountain slopes (grasslands)	*Rheum undulatum* L., *Agropyron michnoi* Roshev., *Arenaria* sp., *Lophanthus chinensis* (Rafin.) Benth., *Pulsatilla turczaninovii* Kryl. et Serg.	*Thalictrum foetidum* L., *Papaver nudicaule* L., *Euphorbia discolor* Ledeb., *Clausia aprica* (Steph.) Korn.-Trotzky	*Agropyron cristatum* (L.) Beauv., *Husluur - Betula* sp.?, *Rhodiola rosea* L., *Orostachys malacophylla* (Pall.) Fisch., *Thymus baicalensis* Serg.
Mountain slopes (with marmot burrow)	*Allium altaicum* Pall., *Morin sharilj - Artemisia* sp.	*Artemisia glauca* Pall. ex Willd.	*Rhodiola rosea* L.
Mountain slope (inner slope)	*Rheum undulatum* L., *Pulsatilla turczaninovii* Kryl. et Serg.	*Sibbaldianthe adpressa* (Bunge) Juz., *Geranium pratense* L., *Thalictrum foetidum* L., *Papaver nudicaule* L., *Euphorbia discolor* Ledeb., *Clausia aprica* (Steph.) Korn.-Trotzky	*Agropyron cristatum* (L.) Beauv., *Agropyron michnoi*Roshev., *Lophanthus chinensis* (Rafin.) Benth., *Rhodiola rosea* L., *Echinops latifolius* Tausch, *Rhaponthicum uniflorum* (L.) DC., *Lilium pumilum* Delile, *Таана - Allium mongolicum* Turcz. ex Regel.
Foothill	*Artemisia frigida* Willd., *Potentilla fruticosa* L., *Allium altaicum* Pall., *Urtica angustifolia* Fisch. ex Hornem., *Pulsatilla turczaninovii* Kryl. et Serg., *Artemisia macrocephala* Jacq. ex Bess.	*Hierochloa* sp., *Gentiana decumbens* L.fill., *Elymus gmelinii* (Ledeb.) Tzvel., *Ranunculus* sp., *Linaria buriatica* Turcz.	*Aconogonon angustifolium* (Pall.) Hara, *Festuca lenensis* Drob., *Echinops latifolius* Tausch, *Sanguisorba officinalis* L., *Lilium pumilum* Delile, *Leontopodium leontopodioides* (Willd.) Beauv.
Shady, uninhabited area			*Cypripedium guttatum* Sw., *Caragana jubata* (Pall.) Poir., *Ribes nigrum* L., *Hyag-Agropyron cristatum* (L.) Beauv.?
Coomb	*Urtica angustifolia* Fisch. ex Hornem., *Potentilla fruticosa* L.		*Echinops latifolius* Tausch, *Delphinium dissectum* Huth, *Betula fruticosa* Pall., *Dianthus superbus* L., *Chamaenerion angustifolium* (L.) Scop., *Iris lactea* Pall., *Trollius asiaticus* L., *Adonis mongolica* Simonovicz, *Lophanthus chinensis* (Rafin) Benth., *Festuca lenensis* Drob.
Shallow coomb	*Rhododendron parvifolium* Adams, *Potentilla fruticosa* L.	*Aegopodium alpestre* Ledeb., *Primula farinosa* L., *Primula nutans Georgi*, *Potentilla nivea* L., *Potentilla* sp., *Cirsium esculentum* (Siev.) C.A.Mey.	*Juniperus pseudosabina* Fisch. et Mey., *Juniperus sibirica* Burgsd., *Parnassia palustris* L., *Galium verum* L., *Hordeum brevisubulatum* (Trin.) Link, *Tsagaan botyuul - Festuca lenensis* Drob.?, *Plantago* sp., *Betula fruticosa* Pall., *Parnassia palustris* L., *Caltha palustris* L.
Near the dung	*Artemisia macrocephala* Jacq. ex Bess., *Rheum undulatum* L., *Morin sharilj - Artemisia* sp.?, *Potentilla anserina* L.	*Leptopyrum fumarioides* (L.) Reichenb., *Shar jaj - Ranunculus* sp.?	*Delphinium dissectum* Huth, *Shavhai nogoon hyag - Agropyron cristatum* (L.) Beauv.?, *Plantago depressa* Schlecht.
Tussocky place along river		*Cirsium esculentum* (Siev.) C.A.Mey.	*Bistorta alopecuroides* (Turcz. ex Meissn.) Kom., *Bistorta vivipara* (L.) S.F.Gray, *Rumex acetosa* L., *Rumex thyrsiflorus* Fingerh.
Edge of the lake		*Glaux maritima* L., *Elymus sibiricus* L., *Puccinellia macranthera* V.Krecz.	*Hordeum brevisubulatum* (Trin.) Link
Warm places (near the winter places)	*Artemisia frigida* Willd., *Urtica angustifolia* Fisch. ex Hornem., *Allium altaicum* Pall., *Rheum undulatum* L.		*Plantago depressa* Schlecht., *Adonis mongolica* Simonovicz
Green meadows	*Potentilla anserina* L.	*Gentiana squarrosa* Ledeb.	*Rumex acetosa* L., *Rumex thyrsiflorus Fingerh*., *Hordeum brevisubulatum* (Trin.) Link
Sayr		*Tsagaan hylgana*?, *Papaver nudicaule* L.	*Thymus* sp., *Lophanthus chinensis* (Rafin.) Benth., *Ranunculus natans* C.A.Mey.
Ruderal place	*Potentilla anserina* L., *Artemisia macrocephala* Jacq. ex Bess.	*Chenopodium album* L.	*Plantago depressa* Schlecht.
Plain steppe in valley	*Artemisia frigida* Willd., *Pulsatilla turczaninovii* Kryl. et Serg.		*Agropyron cristatum* (L.) Beauv., *Agropyron michnoi* Roshev., *Oxytropis pseudoglandulosa* Gontsch. ex Grub., *Plantago depressa* Schlecht., *Gentiana decumbens* L.fill., *Iris lactea* Pall., *Orostachys malacophylla* (Pall.) Fisch.

#### Other salient species groups

There were some species and species groups that were often used to describe habitat types (see above: *botyuul*, *hyag*, *shireg*, *borog*). Most of these had high (or low) forage quality. Herders know these species for example by tasting them. One herder said: *a good herder tastes all the grasses on his pasture*.

There were two species groups locals also often mentioned: hot and cold grasses and leafy plants.

Hot grasses are *leafy, they are of high quality*, and *they stay in the livestock*, while cold grasses *only fill the stomack*, and *go through the livestock* (without much effect on meat or milk production). They argued that *livestock should eat hot grasses in the summer to fatten well*. Hot grasses are, for example, *Artemisia frigida* Willd., *Oxytropis pseudoglandulosa* Gontsch. ex Grub., *Pedicularis* spp., *Agropyron cristatum* (L.) Beauv., *Pulsatilla turczaninovii* Kryl.et Serg., *Pulsatilla flavescens* (Zucc.) Juz., and *Echinops latifolius* Tausch.

Leafy plants have *large leaves*, and their most salient feature is that *they are trampled and destroyed by livestock easily in autumn, while grasses are less* impacted. Examples are *Gentiana macrophylla* Pall., *Plantago depressa* Schlecht., *Vicia amoena* Fisch., *Pedicularis resupinata* L., *Valeriana officinalis* L., *Aegopodium alpestre* Ledeb., *Sanguisorba officinalis* L., *Hedysarum alpinum* L., *Rumex acetosa* L., and *Trollius asiaticus* L*.* They have various nutritional value.

## Discussion

Mongolian herders had a deep knowledge of the habitats of their landscape and of the habitat preferences of the 76 plant species interviewed. They distinguished altogether at least 88 folk habitats. Folk habitat categories were more or less discrete units, perceptually and functionally distinct elements, but often had diffuse boundaries towards neighboring types (cf. [21, 22]). Geomorphologically defined habitats had the highest number of categories, followed by forests, wetland, and rock habitats.

### Naming of folk habitats

Many folk habitat names were shorter or longer descriptive expressions often using common words (e.g., wet meadow with tussocks along stream; dampy shallow coomb on the nortern slopes with bushy *pea shrub* and willow; flint rocky place with sand and low grass). This might indicate flexibility (cf. [43]) and the lack of a fixed terminology for many habitat types listed in Tables 2, 3, 4, 5, 6, 7, 8, and 9. In other cases, habitats had a short and specific name (e.g., *taiga*, *Gobi*, *tsaram*, *shireg*, *borog*), and people understood the same under these names. Hunn and Meilleur [21] suggest that these habitats and names provide the basic level of the local landscape partitioning—landscape elements which are particularly salient to the local community. Molnár [16, 20, 52] reports that a large number of synonyms are attributable to the erosion of knowledge, limited knowledge sharing, and diverse ethnic origins of locals. In contrast, in other landscape partitionings [14, 22, 53, 54], categories are highly lexicalized and have only few synonyms.

Several habitat terms were locative, especially in the geomorphological and hydrological sets (e.g., among the trees), and were literally the same as those used by the Csángós in the Carpathians [22], by the Matsigenka in Amazonia [14], and by the Gitksan and Kaska in Canada [39, 40].

Habitat names did not reflect directly the usefulness of the habitat (e.g., forage quality), but during the interviews, locals often described the nutritional quality and palatability of the vegetation in the various habitats. The livelihood of the local community had an impact not only on the knowledge of various plants species, but on the local knowledge of habitats as well, they usually described habitats through the eye of their livestock (cf. [12, 42, 52, 55]).

### Scale of folk habitats

Herders distinguished macro-, meso-, and micro-scale habitats (sensu [22]). One reason for the use of different spatial scales may be ecological, since plant species occupy somewhat different niches in a landscape: some species are specialist, while others are generalist—occurring in various habitats in the landscape. For a precise description of species-specific habitat preferences, a multi-scaled landscape partitioning might be better suited (cf. [22]). We also found, just as previous studies had observed [14, 20, 22, 44, 56] that abiotic features (e.g., geomorphology, hydrology, edaphic conditions) often defined larger, broader habitat categories, while biotic features defined habitats were used in the finer-scale partitioning.

Most habitats (77%) belonged to the meso-scale, while macro-scale and micro-scale habitats were few; however, research methodology (less participatory field work) might be partly responsible for this.

Herders’ perceptions of spatial variability in their environment are reflected in their nomadic herding strategy [3]. A key division of the landscape is by partitioning it into winter and summer places (pastures). Summer places—where the main goal is fattening and rearing youngs—are chosen by ecological criteria including availability of water for livestock and domestic use and availability of optimal forage types for the different kinds of livestock (cf. [3, 50]), while winter places serve to keep the condition of the livestock as far as possible till the spring and are chosen based on available forage and wind and snow conditions.

Herders distinguished two major parts in the summer place: inner and behind (outer) parts, former one being closer to the valley bottoms and yurts (for a similar partitioning, see [22]). However, we could not find a sharp distinction between primary (hardly human-transformed) habitats and semi-natural/agricultural habitats which is a well-documented basic dichotomy of the folk habitat classification systems of several tropical peoples (e.g., primary forest vs. secondary forest in swidden systems) [14, 44, 53]. In our study area, cultivated lands and other drastically transformed habitats are totally missing. The main reason for this is probably rooted in the traditional beliefs of Mongols (and most of Inner-Asian nomads); human beings have no right to change anything in their surrounding, any change should happen spontaneously, naturally.

### Dimensions of the Mongolian landscape partitioning

Mongolian habitat categories were not organized into a single hierarchy; the partitioning was multidimensional. The multidimensional description of habitats incorporated several sets of features and made the nuanced characterization of plant species’ habitat preferences possible.

Based on our understanding, exposure of slopes and valleys in between gave the major structure to the classification. Otherwise, Mongolians distinguished habitats by the following features (Tables 3, 4, 5, 6, 7, 8, 9, and 12): geomorphology, dominant plant species, soil and bedrock types, hydrological features, topographic features, vegetation structure, successional stage, land use, and disturbances. Fernández-Giménez [3] also found that Mongolian herders classify pasture habitats using a diverse set of criteria, including nutritional quality, topography and elevation, aspect, ecological zone and plant community, color, soil characteristics, water quality and quantity, distance from camp, and degree of utilization by livestock.

**Table 12:** Dimensions of habitat classifications and landscape partitionings in the temperate-boreal regions in Northern Eurasia and North America

Dimensions	Gitksan and Kaska people, W-Canada [15, 19, 39]	Alleutais community, French Alps [41]	Hortobágy herders, Hungary [16, 20]	Csángó community in the Carpathians [22]	Herders in Arbulag soum, Mongolia (recent study)
Edaphic dimension (bedrock, soil)	**	***	***	**	***
Hydrology (wetness)	***	***	**	**	**
Geomorphology (slope, aspect)	***	***	**	**	***
Topography (elevation)	**	**	*	*	*
Vegetation structure (physiognomy)	**	*	**	***	**
Dominant plant species	**	**	***	***	**
Game and other wild animals	**	*	*	*	*
Succession (stages)	*	*	**	**	*
Disturbance (natural and human)	**	**	**	***	*
Land use	*	***	***	***	*

Asterisks indicate how important certain dimensions may be in the local habitat systems

In other parts of the world but in similar mountainous environments like in the Carpathians [22] and the Alps [13, 41, 57], locals use similar features in the recognition and naming of habitats to partition their landscapes (Table 12). Johnson [39, 40] also documented the importance of physiography, hydrologic features, vegetation, and also wildlife habitats among Gitksan and Kaska Dena First Nations in Western Canada. In the European mountainous landscapes, especially in the Carpathians, land use seems to be the key dimension, while all other habitat categories are actually refining the basic land-use types—forests, haymeadows, pastures, and arable fields [13, 22, 42].

On the contrary, in geomorphologically more simple landscapes, habitats can be defined by various abiotic and biotic factors that are arranged along a key gradient (very gently slope with changing soil quality, i.e., dimensions highly correlate), while many possible gradients (e.g., woody/non-woody, mountain/valley, rock/sand, naturalness) can be missing [Hortobágy: 20, 52; Amazonia: 56].

Vegetation (and vegetation-dominated habitats) can be classified in many ways. Methodologies are based on species composition (see, e.g., the most commonly used scientific so-called Braun-Blanquet phytosociological approach [17]), vegetation physiognomy, vegetation structure, or environmental factors. Mongolian herders never used species composition (list of characteristic and dominant plant species) as one of their features, similarly to Csángó people and Hortobágy herders [20, 22]. Mongolians used edaphic, hydrological, vegetation, structural, etc. features instead.

In summary, local landscape partitionings differed considerably in the importance of various dimensions used, with edaphic, geomorphological, hydrological, and dominant species-based dimensions having higher, while topographical, successional, and zoological dimensions having lower importance.

Geomorphologically defined habitats (like slopes, ridges, valleys) were common in our study area. Reasons behind this high number might be that (1) the landscape has a diverse geomorphology, (2) the different habitat types have very different usability (e.g., forage value), and (3) geomorphological features play an important role in orientation (e.g., routes for long-distance travels).

Aspect (exposure) was a crucial feature. Southern and northern slopes were sharply distinguished. In the Northern Mongolian forest steppe area, forests almost exclusively occur on northern slopes, while southern slopes dominated by meadow steppes [58] provide high-quality forage for the livestock (cf. hilly and mountainous temperate landscapes in the northern hemisphere [22, 39]).

Dynamic aspects, like changes in vegetation and other habitat features, were also mentioned several times. Forest changes are slow in this region partly because of very slow tree growth rate, partly because forest edge trees are protected both by local customs and the state from cutting [59]. The habitats “young forest,” “old forest,” and “dying forest” indicated the understanding and importance of forest regeneration and succession, but forest use was less intensive, and so, the sequence was much less detailed than in the Carpathians [22]. Shrub habitats were few, probably because most shrub species are grazing intolerant and are rare. Screes serve as refuges for several shrub species and herders often assigned shrub species to these habitats.

Herders were aware that grassland dynamics is slow partly because of the dense root system (the roots of pasture plants *rule the earth*, *the vegetation is saturated*). The fastest grassland succession was observed by herders on places on recently abandoned yurt and pen sites that were also called *lifeless soil* (documented as “death of the soil” by Fernández-Giménez [3]).

Mongolians like to exist in the landscape in a way causing as little disturbance as possible. This may be one of the reasons why heavily disturbed places occupy small areas; vegetation stands with fast successional dynamics are less common than in other landscapes in the temperate region. Csángó people in the Carpathians also do their best to minimize disturbance and/or facilitate regeneration as effectively as possible during farming (though farming implies a complex set of deliberate and undeliberate disturbances) because disturbances may reduce the amount and/or quality of hay biomass, which they try to avoid. This might be a reason why disturbance is a salient feature in their landscape partitioning [22].

Herders often mentioned salient plant species and morphologically or ecologically salient indicator species when describing habitat types or habitat preference of other species (see also [24, 56, 60]). Most specialist and generalist species regarded as such by local herders were regarded so by us as well. Local and scientific understandings seemed to correlate. The majority of indicator species were woody in our study area [similarly to [54, 56], but see [22], with primarily herbaceous plants].

## Conclusions

A good Mongolian herder is said to constantly monitor both his herds and his pastures, seeking to “harmonize” the needs of his stock with temporal changes in plants, weather, and water availability [3]. Our informants had the same view and explained to us that *the herder does not follow the livestock* (passively), *it is his duty to think* (about options) *and move them* (to the next right place to graze or rest). They also argued that *herders have to be observant*, for example, tasting all important forage species of their pastures and work for the well-being of their animals. The rich traditional habitat knowledge we were able to document proves that many herders are observant and have a strong dedication to understand the landscape which provides their everyday livelihood.

We conclude that despite all the difficulties of studying landscape partitionings, conducting landscape ethnoecological research will hopefully contribute to a deeper understanding of how nature is perceived by local people at the landscape level. Folk habitat knowledge and, in general, locals’ ecological understanding seem to be a rich information source about the landscape, which can be used in resource management and nature conservation (cf. [61]). We hope that our study can help various stakeholders working at different governance levels and being responsible for a better management of Mongolian pasture resources, and thus, governance can better support small-scale Mongolian herders and their families. This is what—among other things—our local herder colleagues asked us to tell the world.

## Data Availability

Voucher specimens for species were deposited in the herbarium of the Institute of General and Experimental Biology of the Mongolian Academy of Sciences.
